# Double diabetes as an effect modifier for adverse perinatal outcome in pregnant women with type 1 diabetes mellitus – a retrospective multicenter cohort study

**DOI:** 10.3389/fendo.2023.1215407

**Published:** 2023-07-28

**Authors:** Aneta Malinowska-Polubiec, Agnieszka Zawiejska, Ewa Romejko-Wolniewicz, Grzegorz Poprawski, Iwona Towpik, Jacek Brązert, Zuzanna Handziuk, Krzysztof Czajkowski

**Affiliations:** ^1^ 2^nd^ Department of Obstetrics and Gynecology, Medical University of Warsaw, Warsaw, Poland; ^2^ Department of Medical Simulation, Chair of Medical Education, Poznan University of Medical Sciences, Poznan, Poland; ^3^ Oncological Gynecology Department, Poznan University of Medical Sciences, Poznan, Poland; ^4^ Department of Internal Medicine, Diabetology and Endocrinology, University of Zielona Gora, Zielona Gora, Poland; ^5^ Department of Reproduction, Poznan University of Medical Sciences, Poznan, Poland

**Keywords:** pregnancy, obesity, metabolic syndrome, neonatal outcome, maternal outcome

## Abstract

**Introduction:**

Double diabetes (DDiab) is defined as T1DM coexisting with insulin resistance (IR), metabolic syndrome (MetS), and/or obesity. Little evidence is available regarding how frequent DDiab is among T1DM pregnancies and whether it affects the perinatal outcome in this population.

**Aims of the study:**

To explore the prevalence of DDiab in early pregnancy in the cohort of pregnant women with T1DM and to examine the association between an early-pregnancy DDiab status and fetomaternal complications characteristic for T1DM in pregnancy.

**Material and methods:**

A retrospective data analysis of the multicenter cohort of N=495 pregnant women in singleton pregnancy complicated with T1DM followed from early pregnancy until delivery in three tertiary referral centers. DDiab status was defined as T1DM plus pre-pregnancy obesity defined as BMI≥30 kg/m^2^ measured at the first antenatal visit (DDiabOb), or T1DM plus pre-pregnancy IR defined as eGDR (estimated Glucose Disposal Rate) below the 25^th^ centile for the cohort measured at the first antenatal visit (DDiabIR). Proportions of the adverse pregnancy outcomes were compared between DDiabOb and Non-DDiabOb and between DDiabIR and Non-DDiabIR patients.

**Characteristics of the study group:**

(data presented as mean(SD) or percentage): age: 30.0(5.1) years; age when T1DM diagnosed: 17.5(8.5) years; T1DM duration: 12.0(7,9) years; microvascular complications (White classes R,F,RF): 11.9%, pre-pregnancy counselling: 26.6%, baseline gestational age: 10.5(4.3) weeks, pre-pregnancy BMI: 23.7(4.3) kg/m^2^; chronic hypertension: 9.1%, gestational hypertension (PIH) 10.7%, preeclampsia (PET): 3.2%; nulliparity 53.8%, smoking in pregnancy: 4.8%, eGWG: 22.4%, DDiabOB: 10.1%; DdiabIR: 25.2%; LGA: 44.0%, and NICU admission: 20.8%.

**Results:**

(data from the univariate analysis given as OR(95%CI)): both DDiabOB and DDiabIR status increased the risk for eGWG [23.15 (10.82; 55.59); 3.03 (1.80; 5.08), respectively]. DDiabIR status increased the risk for PET [4.79 (1.68;14.6)], preterm delivery [1.84 (1.13; 3.21)], congenital malformation [2.15 (1.07;4.25)], and NICU hospitalization [2.2 (1.20;4.01)]. Both DDiabOB and DDiabIR accurately ruled out PET (NPV 97.3%/98.3%, accuracy: 88.3%/75.6%, respectively), congenital malformation (NPV 85.6%/88.4%, accuracy: 78.9/69.8, respectively), and perinatal mortality (NPV 98.7%/99.2%, accuracy: 88.8%/74.5%, respectively).

**Conclusions:**

Double diabetes became a frequent complication in T1DM pregnant population. Double diabetes diagnosed in early pregnancy allows for further stratification of the T1DM pregnant population for additional maternal risk.

## 1 Introduction

Double diabetes (DDiab) is defined as type 1 diabetes (T1DM) coexisting with insulin resistance (IR), metabolic syndrome (MetS), and/or obesity (1). This new concept addresses a dual burden of type 1 diabetes and obesity, which affects a disproportionately large proportion of the T1DM population (2).

The complications of diabetes affecting the mother and fetus are well-known: pre-eclampsia, nephropathy, fetal wastage from early pregnancy loss or congenital anomalies, macrosomia, birth trauma, shoulder dystocia, cesarean section, preterm labor, postoperative wound complications, stillbirth, and neonatal hypoglycemia. Pregnant women with obesity are at increased risk for maternal and perinatal complications, and the risks are amplified with increasing severity of the condition (3–5). It has been estimated that one-quarter of pregnancy complications (e.g. gestational hypertension, preeclampsia, gestational diabetes, preterm birth, large for gestational age [LGA] infant) are attributable to the mother being obese or overweight (5). Patients with pre-pregnancy obesity followed by high gestational weight gain have the highest risks of pregnancy complications. Offspring of pregnant mothers with obesity are at increased risk of developing obesity in childhood and as adults (6, 7). The presence of obesity among T1DM pregnant women is expected to aggravate these complications.

Little evidence is available regarding how frequent DDiab is among T1DM pregnancies and whether it is related to the perinatal outcome in this population. Existing literature is scarce and addresses perinatal outcomes in T1DM pregnant women with concomitant obesity (8–10), while single pieces of evidence attempt to explore metabolic syndrome in this population (11). This body of evidence consistently reported associations between increased maternal BMI and large for gestational age birth weight, NICU admissions, and congenital malformations (8–10).

Insulin resistance is difficult to measure in individuals without endogenous insulin excretion. A small body of evidence explored surrogate markers for insulin resistance applicable in subjects with type 1 diabetes mellitus, the so-called estimated glucose disposal rate (eGDR). The eGDR score was originally developed and validated with the euglycemic-hyperinsulinemic clamp in a subset of 24 participants with T1D from the Pittsburgh EDC study (12). These authors initially calculated eGDR using clinical factors including waist–hip ratio (WHR), presence of hypertension, and HbA1c; however, they also stated that replacing WHR with either BMI or waist circumference provided a similar association with insulin resistance (12–14). There is no available data on maternal eGDR and its association with perinatal outcomes in pregnant women with T1DM.

Therefore, we designed the retrospective cohort study to explore the prevalence of double diabetes measured as coexistent pre-pregnancy obesity or early pregnancy insulin resistance, in the cohort of women with T1DM, and to examine the associations between an early-pregnancy DDiab status and feto-maternal complications characteristic for T1DM in pregnancy. We hypothesized that double diabetes could be an effect modifier changing the effect size for the association between maternal T1DM and adverse maternal or fetal complications characteristic for this population.

## 2 Material and methods

Our study presents the results of the retrospective analysis of clinical data routinely collected between 2015 and 2022 in three public perinatal tertiary referral centers that provide antenatal care for pregnant women with type 1 diabetes mellitus (T1DM).

All women referred to the centers in early pregnancies complicated with T1DM were considered eligible for the study. Multiple pregnancies, early pregnancy loss, or participants lacking information necessary to diagnose double diabetes using at least one criterion (see below) were excluded from the analysis. Finally, data from N=495 individuals were included in the analysis. The following information was retrieved from medical records: patients’ age, age upon the DM diagnosis, duration of the disease, gestational age upon the first visit to the center, pre-pregnancy counselling, history of vascular complications (microvascular complications, White classes: R, F, R/F), parity, smoking status in early pregnancy, data regarding body weight and height, long-term metabolic control expressed as HbA1c measured at the first antenatal visit in the referral center, history of hypertensive disorders of pregnancy, gestational weight gain, and neonatal outcome.

Hypertensive disorders of pregnancy (HDP) were defined as chronic hypertension, gestational hypertension, or preeclampsia (15). Gestational weight gain (GWG) was defined as a difference between the maternal body weight recorded during admission for delivery and maternal body weight recorded at the first antenatal visit in the first trimester of pregnancy (gestational age below ten weeks). Excessive GWG (eGWG) was defined as the GWG above the recommendations set by the LifeCycle Consortium (16).

Information regarding neonatal outcome included gestational age at delivery, birthweight, neonatal sex and information about congenital malformation, neonatal hypoglycemia, phototherapy, NICU (Neonatal Intensive Care Unit) admission, and neonatal mortality defined as stillbirth or intrapartum/neonatal death. Large-for-gestational age birthweight (LGA) was defined as a birth weight equal to or above the 90^th^ percentile according to the Intergrowth21 growth chart (17). Small-for-gestational age (SGA) birthweight was defined as a birth weight equal to or below the 10^th^ percentile according to the Intergrowth21 growth chart (17).

Two methods were used to diagnose double diabetes (DDiab) in the cohort. DDiabOB was defined as T1DM coexisting with pre-pregnancy obesity expressed as BMI ≥30 Kg/m^2^ measured at the first antenatal visit. DDiabIR was defined as T1DM coexisting with pre-pregnancy insulin resistance, expressed as the estimated glucose disposal rate (eGDR) within the lowest quartile, measured using the formula developed by Helliwell et al. for the population with T1DM (18).

Estimated glucose disposal rate (eGDR) has been proposed as an alternative method to measure insulin resistance in individuals with type 1 diabetes mellitus, which is easy to apply in clinical settings. The formula used for eGDR calculation are shown below (18):


$$\text{eGDRBMI}=19:02-(0.22\times \text{BMI [kg}/{\text{m}}^{2}]-(3.26\times \text{HTN})-(0.61\times \text{HbA}1\text{c}[\%])$$

where HTN is hypertension (1 = yes, 0 = no).

Statistical analysis was performed with R version 4.2.2 (19) and MedCalc^®^ Statistical Software version 20.111 (MedCalc Software Ltd, Ostend, Belgium; https://www.medcalc.org; 2022). Incidences of maternal or neonatal outcomes were compared between DDiabOB and non-DDiabOB individuals, and between DDiabIR and non-DDiabIR individuals. Univariate logistic regression was used to explore whether DDiabOB or DDiabIR status is associated with changes in odds for any of the studied outcomes. We examined whether the associations remained significant in multivariate models after controlling for the prespecified covariates. We also examined diagnostic properties of the DDiabOB or DDiabIR status in early pregnancy for predicting maternal or neonatal adverse outcomes, calculating sensitivity, specificity, positive predictive value, negative predictive value, and accuracy. Finally, we analyzed ROC curves to explore how well early pregnancy eGDR and maternal BMI predict maternal or neonatal complications in this cohort. *p*<0.05 was considered statistically significant.

## 3 Results

**Table 1** summarizes the characteristics of the study group and the maternal outcome stratified by the DDiabOb or DDiabIR status. Our patients were predominantly nulliparous. Approximately 12% had microvascular complications diagnosed before the pregnancy. Only one-quarter of the cohort were pre-pregnancy counselled. We recorded a high incidence of hypertensive disorders of pregnancy, complicating over twenty per cent of the cohort. Data about treatment mode were available for 97.6% (N=483) of subjects: 50.9% were on CSII (continuous subcutaneous insulin infusion; insulin pump), whereas the remainder were treated with MDI (multiple daily injections; basal-bolus therapy).

**Table 1:** Maternal characteristics of the study group; *data expressed as mean (SD) or percentage*.

	N=495	T1DM+obesity			T1DM+insulin resistance		
		DDiabOB N=50	non-DDiabOB N=444	*p*	DDiabIR N=103	non-DDiabIR N=306	*p*
Age (years)	30.0 (5.1)	31.6 (4.6)	30.0 (5.2)	*0.022*	30.6 (4.9)	29.9 (5.1)	0.202
Age when diagnosed with T1DM (years)	17.5 (8.5)	18.8 (9.3)	17.4 (8.4)	0.372	15.8 (8.1)	17.9 (8.6)	*0.040*
Duration of the disease (years)	12.0 (7.9)	13.2 (9.4)	12.6 (7.8)	0.705	14.5 (7.6)	12.2 (7.9)	*0.017*
Gestational age upon the first visit to the center (gestational weeks)	10.5 (4.3)	10.1 (3.4)	10.6 (4.4)	0.432	10.1 (3.3)	10.3 (3.9)	0.604
Pre-pregnancy BMI (kg/m2)	23.7 (4.3)	32.7 (3.0)	22.7 (2.9)	*<0.0001*	28.2 (5.2)	22.3 (3.0)	*<0.0001*
Early pregnancy eGDR (mg*kg^-1^*min^-1^)	9.48 (1.74)	7.0 (2.08)	9.79 (1.42)	*<0.0001*	7.1 (1.4)	10.2 (0.9)	*<0.0001*
Early pregnancy HbA1c [%]	6.6 (1.3)	6.8 (1.4)	6.6 (1.3)	0.378	7.5 (1.6)	6.3 (1.0)	*<0.0001*
DDiabOB (%)	10.1	–	–	–	37.9	2.0	*<0.0001*
DDiabIR (%)	25.2	86.7	17.6	*<0.0001*	–	–	–
GWG (kg)	13.3 (5.9)	12.1 (8.8)	13.5 (5.5)	0.331	12.9 (7.1)	13.6 (5.6)	0.413
Pre-pregnancy counselling (%)	26.6	13.3	28.1	0.127	19.0	30.5	0.069
Nulliparity (%)	53.8	42.0	55.1	0.108	44.7	57.7	*0.029*
Microvascular complications (White classes: R, F, R/F) (%)	11.9	14.0	11.7	0.645	14.6	11.8	
Smoking in pregnancy (%)	4.8	8.9	4.4	0.258	4.2	4.3	1.00
Chronic hypertension (%)	9.1	24.0	7.5	*0.0007*	37.9	1.3	*<0.0001*
Gestational hypertension (%)	10.7	10.0	10.8	1.00	8.7	9.8	0.893
Preeclampsia (%)	3.2	10.0	2.7	0.07	8.7	2.0	*0.004*
Hypertensive disorders of pregnancy, overall (%)	20.2	34.0	18.7	*0.018*	46.6	11.8	*<0.0001*
Excessive GWG (%)	22.4	81.8	16.3	*<0.0001*	39.6	17.8	*<0.0001*

eGDR, estimated glucose disposal rate; BMI, body mass index; GWG, gestational weight gain; T1DM, diabetes mellitus type 1; DDiabOB, T1DM plus pre-pregnancy obesity; DDiabIR, T1DM plus pre-pregnancy insulin resistance.

Italic values are statistically significant.

**Table 2** summarizes neonatal outcomes in the total cohort stratified by the DDiabOB or DDIabIR status. Neonatal outcomes did not differ by the DDiabOB status. On the contrary, patients with DDiabIR delivered significantly earlier and had larger proportions of premature deliveries, congenital malformations, and NICU admissions compared to the non-DDiabIR participants.

**Table 2:** Neonatal outcome in the cohort; data expressed as mean (SD) or percentage; perinatal mortality given as promille.

	Total cohort N=495	T1DM+obesity			T1DM+insulin resistance		
		DDiabOB N=50	non-DDiabOB N=444	*p*	DDiabIR N=103	non-DDiabIR N=306	*p*
Gestational age at delivery (weeks)	37.1 (2.1)	37.0 (2.3)	37.1 (2.1)	0.877	36.6 (2.3)	37.3 (2.0)	*0.004*
Birth weight (g)	3479 (709)	3640 (755)	3460 (703)	0.115	3499 (857)	3453 (624)	0.616
LGA (%)	44.0	47.0	43.5	0.762	50.0	40.5	0.117
SGA (%)	2.8	0.0	3.2	0.380	3.9	2.6	0.506
Preterm delivery<37 completed gestational weeks (%)	22.6	21.0	22.7	1.00	31.4	19.1	*0.014*
Preterm delivery<34 completed gestational weeks (%)	8.1	6.1	8.4	0.783	10.8	6.6	0.195
Perinatal mortality (%)	12/1000	0.0	13.7/1000	0.89	19.8/1000	6.6/1000	0.261
Congenital malformation (%)	14.5	15.8	14.4	0.810	19.5	10.1	*0.040*
Neonatal hypoglycemia (%)	38.6	42.1	38.3	0.778	44.8	36.6	0.224
Phototherapy (%)	49.7	50.0	49.9	1.00	54.0	46.2	0.286
NICU admission (%)	20.8	21.0	20.8	1.00	33.3	18.0	*0.005*

T1DM, diabetes mellitus type 1; DDiabOB, T1DM plus pre-pregnancy obesity,; DDiabIR, T1DM plus pre-pregnancy insulin resistance LGA, large-for-gestational age birth weight; NICU, neonatal intensive care unit; SGA, small-for-gestational age birth weight; NICU, neonatal intensive care unit.

Italic values are statistically significant.

We used univariate and multivariate logistic regression to examine whether double diabetes (either DDiabOb or DDiabIR) is associated with maternal or fetal outcomes. **Table 3** summarizes significant associations between the double diabetes status in early pregnancy and maternal or neonatal outcomes. In a univariate analysis, DDiabIR was associated with significantly increased risk for PET, excessive GWG, preterm delivery before completed 37 gestational weeks, congenital malformations, and NICU admission. These associations remained statistically significant after controlling for maternal age, disease duration, vascular complications, and pre-pregnancy counselling.

**Table 3:** Double diabetes as a predictor for maternal or fetal complications; data from univariate (crude OR) and multivariate (adjusted OR) logistic regression models.

The outcome	DDiabOB		DDiabIR	
	Crude OR	Adjusted OR^*)^	Crude OR	Adjusted OR^**)^
Preeclampsia	–	–	4.79 (1.68; 14.61)	1.21 (0.22; 6.22)
Excessive GWG	23.15 (10.82; 55.59)	29.0 (9.19; 116.52)	3.03 (1.8; 5.05)	2.60 (1.38; 4.91)
Preterm delivery below 37 gestational weeks	–	–	1.94 (1.16; 3.21)	1.71 (0.91; 3.16)
Congenital malformation	–	–	2.15 (1.07; 4.25)	2.76 (1.31; 5.78)
NICU hospitalization	–	–	2.28 (1.30; 4.00)	2.2 (1.20; 4.01)

^*)^adjusted for age, pre-pregnancy counselling, diabetes duration, vascular complications, chronic hypertension and early pregnancy HbA_1c_; ^**)^adjusted for age, pre-pregnancy counselling, diabetes duration and vascular complications; GWG, gestational weight gain; NICU, neonatal intensive care unit.

**Table 4** summarizes the predictive diagnostic properties of DDiabOB or DDiabIR status in predicting selected maternal or fetal complications. Testing for DDiabOB or DDiabIR status in early pregnancy was an effective rule-out test for eGWG, PET, preterm delivery before completed 37 weeks of gestation, congenital malformations, NICU hospitalization and perinatal mortality, showing high NPV (between 80.3% and 99.2%), and good or acceptable accuracy (between 68.3% and 88.8%). However, maternal pre-pregnancy obesity did not discriminate those with the outcome from those without it if used as a threshold for ROC curves for the following outcomes: preterm delivery before completed 37 gestational weeks, congenital malformations and NICU hospitalization (95%CIs for all these ROC curves contain 0.5).

**Table 4:** Double diabetes status (DDiabOB or DDiabIR) as a diagnostic test for selected maternal or neonatal complications.

	Outcome	Sensitivity	Specificity	PPV	NPV	AUC (95%CI)	Accuracy
DDiabOB	eGWG	34.3	97.8	81.8	83.7	0.66 (0.62; 0.70)	83.6
DDiabIR	eGWG	42.7	80/3	38.4	80.3	0.61 (0.56; 0.66)	71.8
DDiabOB	PET	25.0	95.4	7.9	97.3	0.58 (0.53; 0.62)	88.3
DDiabIR	PET	60.0	76.1	7.7	98.3	0.68 (0.63; 0.77)	75.6
DDiabOB	Preterm delivery<37 gestational weeks	9.9	90.0	22.4	77.4	0.50 (0.45; 0.54)	71.8
DDiabIR	Preterm delivery<37 gestational weeks	35.5	77.8	31.9	80.5	0.57 (0.52; 0.62)	68.3
DDiabOB	Congenital malformation	10.5	90.5	15.8	85.6	0.50 (0.45; 0.56)	78.9
DDiabIR	Congenital malformation	42.5	74.4	22.0	88.4	0.58 (0.53; 0.64)	69.8
DDiabOB	NICU hospitalization	9.8	90.4	21.0	79.2	0.50 (0.45; 0.55)	73.6
DDiabIR	NICU hospitalization	41.2	76.3	31.4	83.2	0.59 (0.53; 0.64)	69.1
DDiabOB	Perinatal mortality	0.0	89.9	0.0	98.7	0.45 (0.40; 0.49)	88.8
DDiabIR	Perinatal mortality	50.0	75.2	2.4	99.2	0.63 (0.58; 0.67)	74.9

eGWG, excessive maternal gestational weight gain; NICU, neonatal intensive care unit; NPV, negative predictive value; PET, preeclampsia; PPV, positive predictive value.

**Table 5** presents ROC curve analysis for early pregnancy eGDR and BMI exploring predictive properties of the continuous covariates. It can be noted that early pregnancy GDR performs better as a predictor for maternal and neonatal outcomes, except for the eGWG, where BMI is a significantly better predictor of the outcome. For all the other outcomes, the difference in the AUC between the surrogate marker for insulin resistance and BMI is not statistically significant.

**Table 5:** Early pregnancy eGDR or BMI as a predictor for maternal or neonatal complications – comparison of the ROC curves.

Outcome	Predictor	AUC (95% CI)	*p* for a difference between the AUCs
Excessive GWG	eGDR	0.64 (0.59; 0.69)	0.022
	BMI	0.70 (0.65; 0.74)	
PET	eGDR	0.68 (0.63; 0.72)	0.612
	BMI	0.62 (0.57; 0.67)	
Preterm delivery<37 gestational weeks	eGDR	0.59 (0.54; 0.64)	0.244
	BMI	0.52 (0.46; 0.56)	
Congenital malformation	eGDR	0.61 (0.55; 0.66)	0.653
	BMI	0.52 (0.47; 0.57)	
NICU hospitalization	eGDR	0.60 (0.55; 0.66)	0.093
	BMI	0.55 (0.50; 0.61)	

GWG,maternal gestational weight gain; PET, preeclampsia; NICU, neonatal intensive care unit.

## 4 Discussion

Our study provides epidemiological data regarding the comprehensive set of outcomes in a large, multicenter cohort of pregnant women with T1DM. Our results provide up-to-date evidence that perinatal risk in the pregnant population with T1DM remains high even though these women enter their pregnancies with good metabolic control and are frequently seen by multidisciplinary teams over their pregnancies. Importantly, we report a high proportion of congenital malformations confirmed immediately after birth, which confirms a complex background of impaired fetal development. We also confirm frequent adverse neonatal outcomes despite using novel therapies such as insulin pumps (CSII) during the pregnancy.

As an analysis of routinely available clinical data, this study also has some limitations. We could not explore socioeconomic determinants of health, which are likely to impact the prevalence of the exposure (maternal pre-pregnancy obesity) and fetomaternal outcomes. Moreover, data regarding glycemic variability recorded from CSII were not routinely available in our cohort. Therefore, we were not able to investigate whether lower HbA1c achieved during pregnancy actually translated into reduced glycemic variability, which would be a desirable treatment outcome likely to mediate the association between lower HbA1c and decreased odds for such maternal complications like excessive maternal gestational weight gain or excessive fetal growth. Finally, our study excluded patients with early pregnancy loss, which might result in underestimation of the prevalence of double diabetes in our cohort.

In our cohort, we explore insulin resistance as an effect modifier for the association between T1DM and adverse pregnancy outcomes, which adds to our study’s novelty. Double diabetes diagnosed in early pregnancy as insulin resistance was related to a high risk of pregnancy complications. Those women with insulin resistance diagnosed at the beginning of the pregnancy had significantly worse glycemic control (expressed as significantly higher HbA_1c_). This may be one of the reasons for the adverse neonatal outcome (delivery before the 37th week of pregnancy, congenital malformation, and NICU admission). Moreover, our study focused on pre-pregnancy/early pregnancy maternal metabolic status, while trends for higher incidence of hypoglycemia/hyperbilirubinemia, LGA, preterm delivery, or NICU admission noted in the DDiabIR arm might be caused by maternal metabolic factors present in the late pregnancy. Our observations are relevant for clinical practice, pointing to the eGDR, according to Helliwell et al. (18) as a useful indicator of pre-pregnancy maternal risk combining all three major risk factors (obesity, poor metabolic control, and hypertension) in the pregnant T1DM population into a single measure. Our observations also confirm the usefulness of this diagnostic tool as a reliable rule-out test for the most severe maternal and fetal complications. Early pregnancy eGDR might also be useful for pre-pregnancy counselling to discuss pregnancy risks with women contemplating pregnancy.

Maternal complications in women with T1DM include preterm labor, pre-eclampsia, nephropathy, birth trauma, cesarean section, and postoperative wound complications. Fetal complications include fetal loss or congenital anomalies, macrosomia, shoulder dystocia, stillbirth, growth restriction if coexisting microvascular complications, and hypoglycemia. Despite evidence showing that short-term complications can be mediated by achieving the desired level of glycemic control during pregnancy (20), our observations confirm that perinatal risk related to early pregnancy remains high even though these women enter their pregnancies with good metabolic control. Data from our cohort confirm that pregnant women with T1DM coexisting with obesity are at additional risk of poor maternal or fetal outcomes. Overall, 75% of patients from our cohort received no pre-pregnancy counselling and 5% admitted to smoking during pregnancy. These findings indicate areas for further improvement in managing such pregnancies outside the routinely recommended improvement in glycemic control. Notably, our results show that our DDiabOB patients differed from their non-obese counterparts regarding smoking in pregnancy (reported almost twice as frequently as among non-obese participants) and lack of pre-pregnancy counselling (received by 13.3% of the DDiabOB women compared to 28.1% of the non-DDiabOB arm). Although the difference was not statistically significant, this observation suggests additional risk of poor maternal or fetal outcomes added to the well-known risks associated with obesity or hyperglycemia.

Baseline BMI in patients with type 1 diabetes used to be lower than population standards before Diabetes Control and Complications Trial (DCCT), probably due to weight loss before diagnosis and suboptimal glycemic control. Current BMI patterns in type 1 diabetes patients mirror those seen in the general population. The relative risk of CVD (cardiovascular disease), CHD (coronary hard disease), stroke, and all-cause mortality continues to be unacceptably high for this population (21). Adverse CVD risk factors associated with weight gain in some participants with type 1 diabetes were seen in the observational data from the EURODIAB cohort, in which 1,800 T1DM patients of reproductive age (mean age 33 years, duration of diabetes 14.8 years, HbA_1c_ 8.2% [66 mmol/mol]) were followed up for 7.3 years (22). Those who gained more than 5 kg over this time had better glycemic control than those with less or no weight gain but also had raised blood pressure and a worse lipid profile. Subgroup analyses of the Diabetes Control and Complications Trial/Epidemiology of Diabetes Interventions and Complications (DCCT/EDIC) study have raised concerns about participants whose weight gain over the follow-up period was associated with increased cardiovascular risk (23). Our study also confirms a disproportionately high cardiovascular risk in pregnant women with DDiab, reporting a 20% incidence of hypertensive disorders of pregnancy, and particularly a high number of PET cases (8.7%) in the DDiabIR patients, which is more than ten times higher than the prevalence expected in this region of the world (24).

In our group, 80% of T1DM pregnant women with obesity and almost 40% with insulin resistance presented with excessive gestational weight gain. Those patients have a relatively high risk of retaining excessive kilograms after pregnancy, which is currently recognized as a risk factor for poor cardiometabolic health in the normoglycemic population (25). In women with T1DM, we need to assume an even more accelerated risk of cardiovascular complications because of the consequences of excessive GWG and postpartum body weight retention, adding to the T1DM burden. Excessive GWG is also a confirmed risk factor for increased neonatal morbidity and offspring obesity in later life in the normoglycemic pregnant population (26). A pilot study by McWhorter et al. performed in a very small cohort of nineteen T1DM pregnant noted that nine out of eleven children born to the T1DM population who exceeded the IOM-recommended GWG became overweight or obese in adulthood (27). These findings remain in line with our observations, indicating that excessive GWG in the T1DM pregnant population is a likely additional, non-glycemic risk factor for neonatal complications, like LGA or neonatal morbidity.

Data from Ferreira-Hermosillo et al. showed that an eGDR< 7.32 mg/kg/min has the highest sensitivity and specificity to detect the presence of metabolic syndrome in patients with T1D (28). Women of reproductive age with double diabetes should be aware of exceptionally high mortality and cardiovascular complication risk (23, 29–31) and the necessity of reducing this risk by addressing modifiable factors (diet, weight gain, intensive treatment, and metabolic control). Although the mean eGDR for our cohort was 9.48 mg/kg/min, DDiabOB pregnant patients had a mean eGDR of 7.0 mg/kg/min. We defined participants within the lowest quartile, i.e. with early pregnancy eGDR below 8.703 mg/kg/min, as having insulin resistance in early pregnancy (DDiabIR). Importantly, with this approach, we got a similar threshold to those used by Helliwell et al. to define insulin resistance in non-pregnant individuals with T1DM (eGDR below 8 mg/kg/min) (18). This finding might indicate that early pregnancy BMI and eGDR could be used as robust proxies to assess the additional pre-pregnancy risk profile in women with T1DM who are considering pregnancy. Indeed, there is substantial overlap between obesity and insulin resistance in our cohort. However, while 86.7% of DDiabOB patients met our criterion for DDiabIR, only 37.9% of DDiabIR subjects were obese. In our understanding, these results show that IR status, defined either as the lowest quartile in the cohort or below the threshold set by Halliwell et al. (18), could be used as a tool to identify a larger population lacking obesity at additional risk, sometimes not easy to identify from the clinical point of view.

Further research is needed to examine the impact of being overweight/obese and insulin resistance before pregnancy in T1DM women on perinatal outcomes and whether the better glycometabolic control before pregnancy and in the first trimester can improve diabetes-related outcomes. Our data also justify calls for interventional trials recruiting women with double diabetes to target obesity and insulin resistance before pregnancy and using modern technologies (like CSII) to examine associations between DDiab and short-term glucose variability. Moreover, exploring insulin resistance in late pregnancy, although challenging in the context of pregnancy-related insulin resistance, could provide novel evidence for driving forces behind such neonatal complications as LGA, hypoglycemia, or hyperbilirubinemia.

## 5 Conclusions

Double diabetes is a frequent complication in T1DM pregnant population. Double diabetes diagnosed in early pregnancy allows for further stratification of the T1DM pregnant population for additional maternal risk.

## Data availability statement

The original contributions presented in the study are included in the article/Supplementary Material. Further inquiries can be directed to the corresponding author.

## Ethics statement

Ethical review and approval was not required for the study on human participants in accordance with the local legislation and institutional requirements. Written informed consent for participation was not required for this study in accordance with the national legislation and the institutional requirements.

## Author contributions

AM-P – study design, data collection and interpretation, and drafting the manuscript. AZ – study design, data analysis and interpretation, and drafting the manuscript. ER-W – study design, data collection and interpretation, and drafting the manuscript. GP – data collection and interpretation. IT – data collection and interpretation. JB – study design. ZH – data collection. KC – study design and supervision of the work.

All authors revised the draft for important intellectual content, approved the final version of the manuscript, and agreed to be accountable for the content of the work.
